# Antineoplastic Activity of 9″-Lithospermic Acid Methyl Ester in Glioblastoma Cells

**DOI:** 10.3390/ijms25042094

**Published:** 2024-02-08

**Authors:** Panagiota Tzitiridou, Vasiliki Zoi, Theodora Papagrigoriou, Diamanto Lazari, Chrissa Sioka, Georgios A. Alexiou, Athanassios P. Kyritsis

**Affiliations:** 1 Neurosurgical Institute, University of Ioannina, 45500 Ioannina, Greece; 2 Laboratory of Pharmacognosy, Division of Pharmacognosy-Pharmacology, School of Pharmacy, Faculty of Health Sciences, Aristotle University of Thessaloniki, 54124 Thessaloniki, Greece; 3 Department of Neurosurgery, University of Ioannina, 45500 Ioannina, Greece

**Keywords:** glioblastoma, gliomas, 9″-lithospermic acid methyl ester, temozolomide, cancer, natural products

## Abstract

To date, many potent compounds have been found which are derived from plants and herbs and possess anticancer properties due to their antioxidant effects. 9″-Lithospermic acid methyl ester is an effective natural compound derived from the *Thymus thracicus* Velen. It has been proven that this compound has substantial properties in different diseases, but its effects in cancer have not been thoroughly evaluated. The aim of this work was to study the effects of 9″-Lithospermic acid methyl ester (9″-methyl lithospermate) in U87 and T98 glioblastoma cell lines. Its effects on cellular viability were assessed via Trypan Blue and Crystal Violet stains, the cell cycle analysis through flow cytometry, and cell migration by employing the scratch wound healing assay. The results demonstrated that 9″-methyl lithospermate was able to inhibit cellular proliferation, induce cellular death, and inhibit cell migration. Furthermore, these results were intensified by the addition of temozolomide, the most prominent chemotherapeutic drug in glioblastoma tumors. Further studies are needed to reproduce these findings in animal models and investigate if 9″-lithospermic acid methyl ester represents a potential new therapeutic addition for gliomas.

## 1. Introduction

Cancer is one of the most common causes of mortality worldwide. Glioblastoma is a type of malignancy that starts with the continuous and uncontrolled growth of cells in the brain or spinal cord [1]. Glioblastoma is formed by glial cells, whose role is to support nerve cells and which can grow fast enough to invade and destroy adjacent tissues [2]. Glioblastoma is the most common malignant primary brain tumor, accounting for 54% of all gliomas and 16% of all primary brain tumors [3]. Glioblastoma is currently a non-curable form of malignancy, with a median survival rate of 15 months [4]. The treatment of glioblastoma is quite complex [5]. It initially involves surgical excision of the tumor followed by radiotherapy combined with the chemotherapeutic agent temozolomide (TMZ) [6]. Even though the combination of radiotherapy with the chemotherapeutic TMZ represents the most effective adjunctive therapy after surgical resection, it only prolongs the survival time for a few months [6]. The treatment of glioblastoma has been a challenge up to this day, indicating that finding new targets is imperative.

In recent years, drugs derived from natural sources (such as plants) have attracted the attention of pharmaceutical research due to their good pharmacological activity, as well as their favorable safety profile [7]. Lithospermic acid [8] and lithospermic acid B [9] are important phenylpropanoid oligomers found in various plants of the Lamiaceae family (commonly known as the mint, nettle, and sage family) and the Boraginaceae family (a family of borage or forget-me-nots that includes about 2000 species of shrubs, trees, and herbs). Lithospermic acid and lithospermic acid B are bioactive constituents in medicinal herbs, including sage (*Salvia officinalis*) and salvia (*Salvia miltiorrhiza* Bunge), which have been used for several centuries in Chinese medicine. Since its first isolation and characterization in 1975, lithospermic acid has been implicated as the main component of many popular plants used in traditional medicine for the treatment of cardiovascular disorders, cerebrovascular diseases, and various types of hepatitis, as well as in chronic renal failure [10]. Research has demonstrated that lithospermic acid and lithospermic acid B possess antioxidant and biological properties, which include the inhibition of the human immunodeficiency virus (HIV) integrase enzyme [11], the inhibition of hyaluronidase [12], and amelioration of uremia symptoms, resulting in significant reductions in blood urea nitrogen, creatinine, and methylguanidine levels [10,13]. It has also been found in an experimental rat model that the use of lithospermic acid B has a preventive effect on the development of diabetic retinopathy, probably due to its antioxidant and anti-inflammatory effects [14], as well as a positive effect during ischemic brain injury [15].

**Figure 1:**
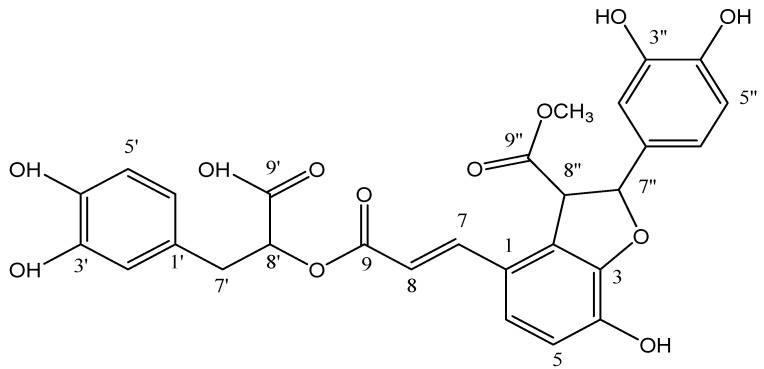
Structure of 9″-lithospermic acid methyl ester.

In the present work, the 9″-monomethyl ester of lithospermic acid (Figure 1), which was isolated from the methanolic extract of *Thymus thracicus* Velen (thyme), was used. Although this compound possesses strong antioxidant properties, it has not been studied for anti-cancer properties. In the present study, we attempt to elucidate its possible antineoplastic activity in glioblastoma cell lines (T98 and U87).

## 2. Results

### 2.1. Sensitivity of Glioblastoma Cells to 9″-Lithospermic Acid Methyl Ester and IC_50_ Calculation

**Figure 2:**
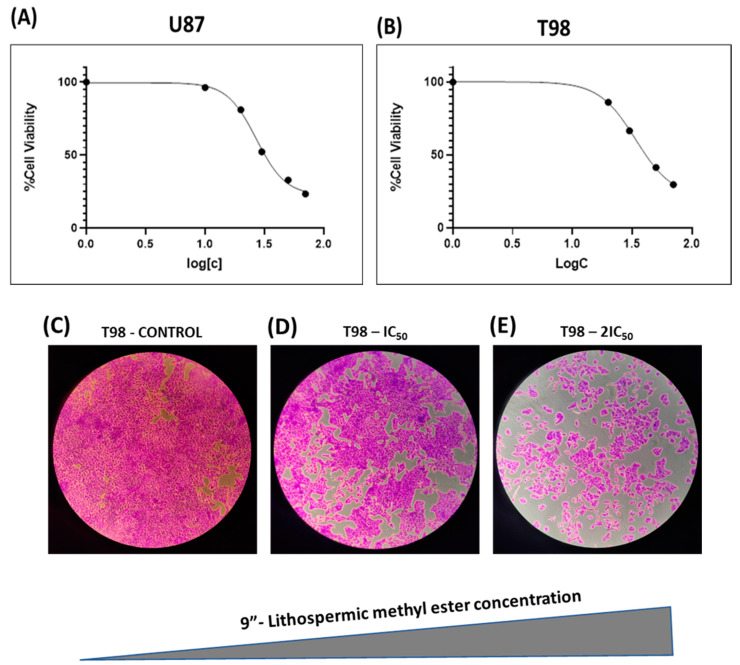
Viability of glioblastoma cells after the treatment with the chemical agent 9″-lithospermic acid methyl ester. The cell viability was assessed via the use of the Trypan Blue exclusion assay (**A**) in the U87 cell line and (**B**) in the T98 cell line. Photos from the optical microscope that show living cells which maintain their ability to attach themselves to the plastic (**C**) without the chemical agent, (**D**) with a concentration equal to IC_50_ = 34 μM, and (**E**) with double the concentration of IC_50_ = 68 μM in the T98 cell line. Images were recorded at 5× magnification.

To determine the anti-cancer activity of 9″-lithospermic acid methyl ester against glioblastoma, U87 and T98 cells were incubated with increasing concentrations (0, 10, 20, 30, 50, and 70 µM) of the chemical agent for 72 h. Using the Trypan Blue exclusion assay, which can separate live from dead cells, and using a hemocytometer, cells were counted at all concentrations, and the percentage of viability, as well as the concentration responsible for 50% of cell death, IC_50_, was determined (Figure 2A,B). This toxicity was confirmed by the assay based on Crystal Violet, which can stain living cells that retain the ability to adhere, in the T98 cell line for concentration values equal to the IC_50_, as well as its doubling, and it was found that at increasing concentrations of 9″-lithospermic acid methyl ester, it has a strong cytotoxic effect (Figure 2C–E).

### 2.2. 9″-Lithospermic Acid Methyl Ester Induces Cell Cycle Arrest

**Figure 3:**
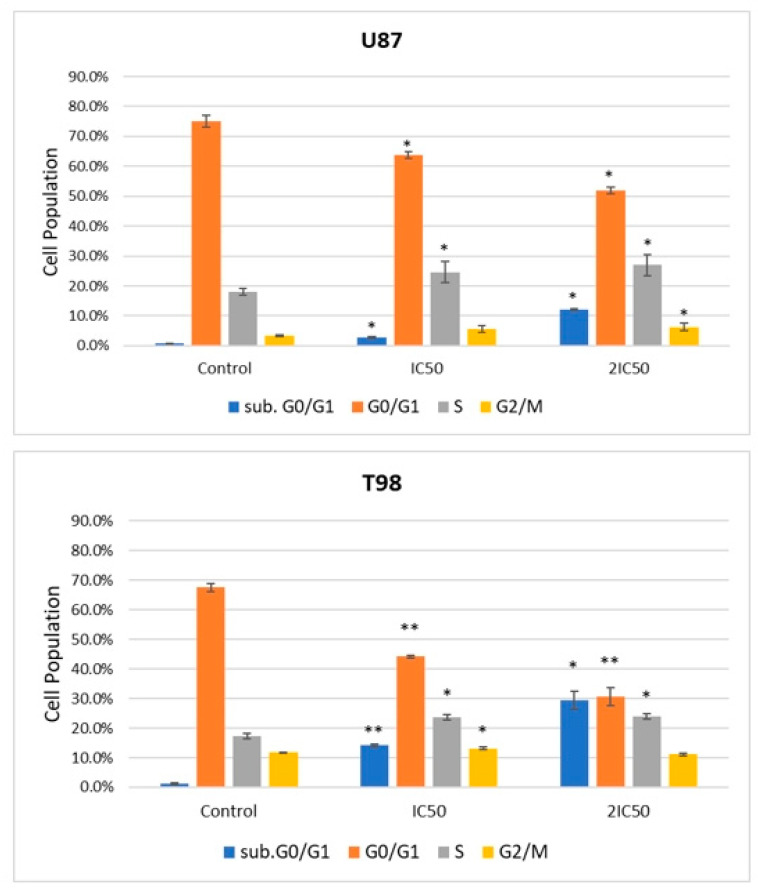
Graphical representation of the cell-cycle phase distribution in cell lines U87 and T98 after treatment with 9″-lithospermic acid methyl ester in IC_50_ and doubled IC_50_ concentrations. Data are expressed as the mean ± SD from three different experiments. * *p* < 0.05; ** *p* < 0.001.

After testing the cytotoxicity of 9″-lithospermic methyl ester in glioblastoma cell lines U87 and T98, the impact of this agent on the cell cycle was investigated. To this end, the U87 and T98 cell lines were incubated for 72 h with concentrations of 9″-lithospermic acid methyl ester equal to IC_50_ and 2IC_50_. Flow cytometry results showed that 9″-lithospermic acid methyl ester led to an accumulation of the cell population in the S phase (from 18.1% to 27% in cell line U87 and from 17.3% to 23.8% in cell line T98) without any change in the subsequent G2/M phase. In addition, there was an increase in the cell population upon incubation with the chemical agent in the sub. G0/G1 phase (from 0.7% to 12.2% in cell line U87 and from 1.2% to 29.3% in cell line T98), an indication of cell death (Table 1 and Figure 3).

### 2.3. 9″-Lithospermic Acid Methyl Ester Inhibits the Migration of Glioblastoma Cells

**Figure 4:**
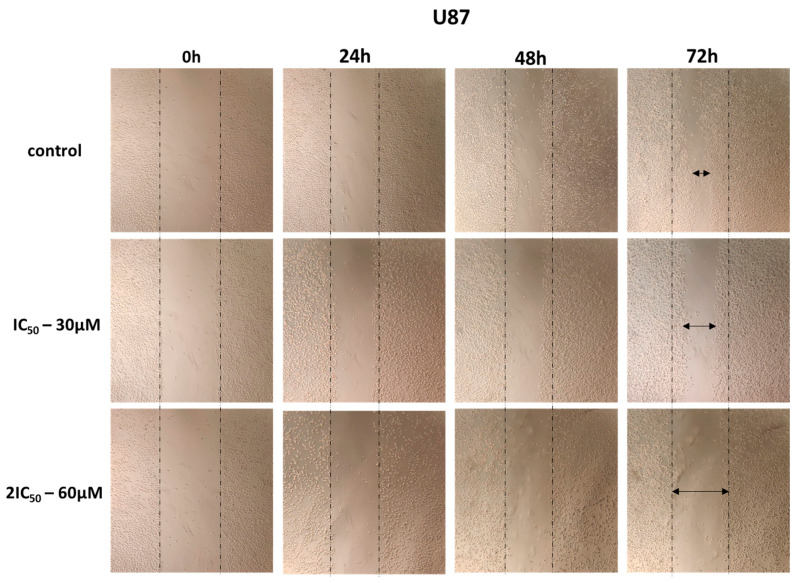
The effect of 9″-lithospermic acid methyl ester on the migration capacity of glioblastoma cell line U87 at 24, 48, and 72 h. The arrows indicate the coverage of the scratch area.

**Figure 5:**
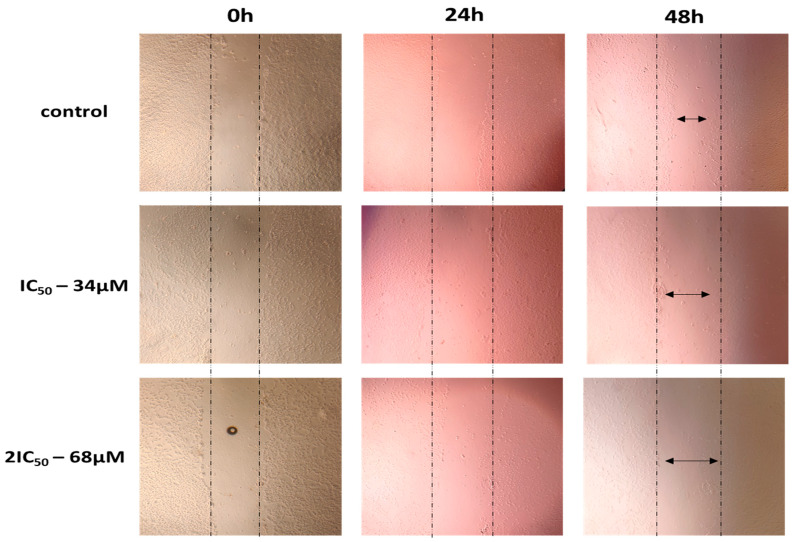
The effect of 9″-lithospermic acid methyl ester on the migration capacity of the glioblastoma T98 cell line at 24 and 48 h. The arrows indicate the coverage of the scratch area.

**Figure 6:**
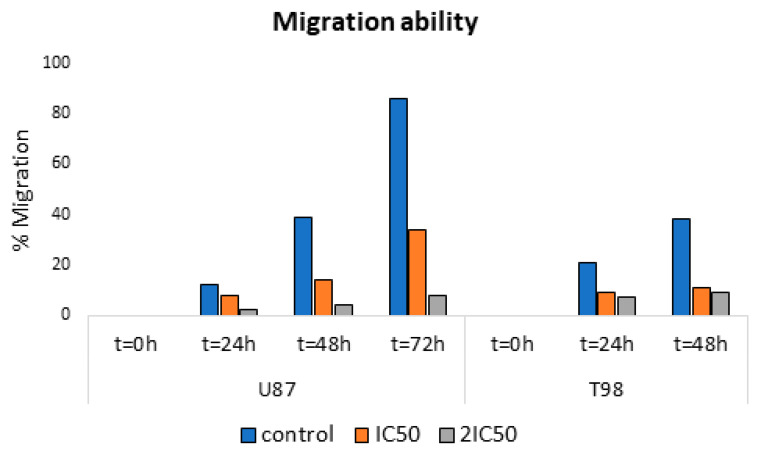
Healing rates of the artificial vacuole in the presence of 9″-lithospermic acid methyl ester in U87 and T98 cells as an average of experiments.

It is known that a characteristic of cancer cells is high motility, resulting in cancer cells leaving the primary tumor and migrating to distant areas. For this purpose, scratch wound healing assay experiments were performed with the chemical agent at concentrations equal to IC_50_ and 2IC_50_, followed by taking pictures at 0, 24, 48, and 72 h. The results showed that the cells, to which the agent was not added, showed high motility resulting in covering the scratch area, whereas incubation with the agent resulted in the inhibition of cell motility and limited coverage of the area (Figure 4, Figure 5 and Figure 6). These results indicate that 9″-lithospermic acid methyl ester inhibited, to a satisfactory extent, the healing of the artificial wound in both cell lines (U87 and T98).

### 2.4. 9″-Lithospermic Acid Methyl Ester and TMZ Exert Synergistic Anti-Proliferative Results

To determine the effect of 9″-lithospermic acid methyl ester in combination with the known chemotherapeutic agent TMZ, cell lines U87 and T98 were incubated at increasing concentrations of these agents. The results were interpreted using the median-effect equation and CompuSyn software (Compusyn vers. 1.0, Inc., Paramus, NJ, USA). The concentrations used were 15, 30, 45, and 60 μM of 9″-lithospermic methyl ester and 25, 50, and 100 μM of TMZ for cell line U87, while concentrations equal to 17, 34, 51, and 68 μM, as well as 200, 300, and 400 μM, were used in cell line T98, respectively. The combination treatment was carried out at different ratios and the combined effect of the two agents was compared with the effect of each individual agent separately. The results of the effect of these two agents are summarized in Table 2.

The combined effect of 9″-lithospermic acid methyl ester and TMZ on U87 cells showed that these two agents exhibit synergy, which becomes more pronounced at higher concentrations. On the other hand, in the T98 cell line, synergy of these factors is observed when administered at low concentrations, whereas at higher concentrations, antagonism is observed. One explanation is that the administration of two different agents may alter the metabolism of the other and thus lead to incomplete release.

**Figure 7:**
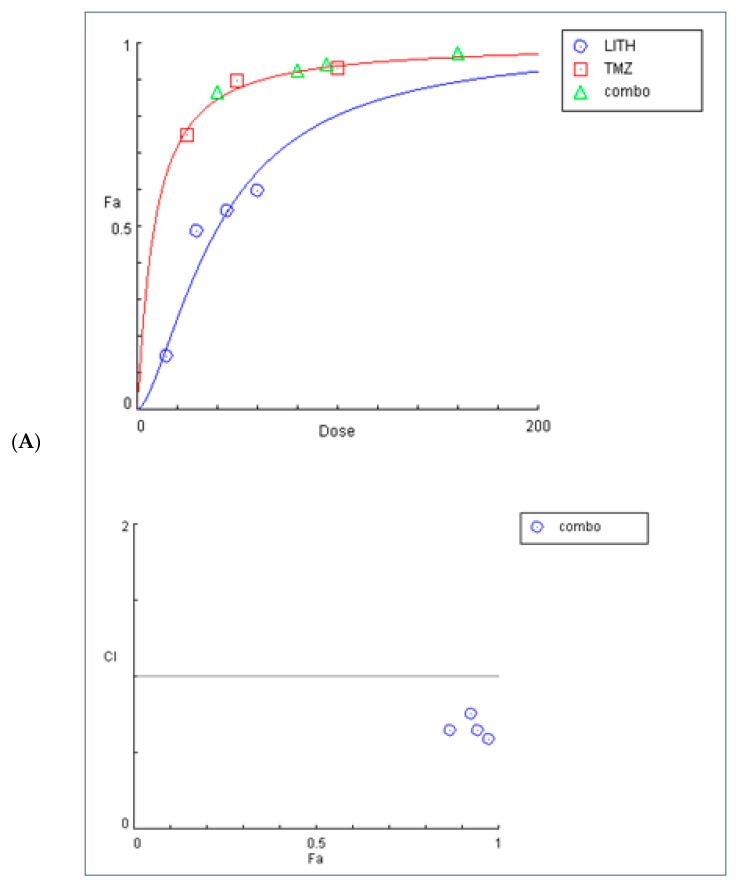

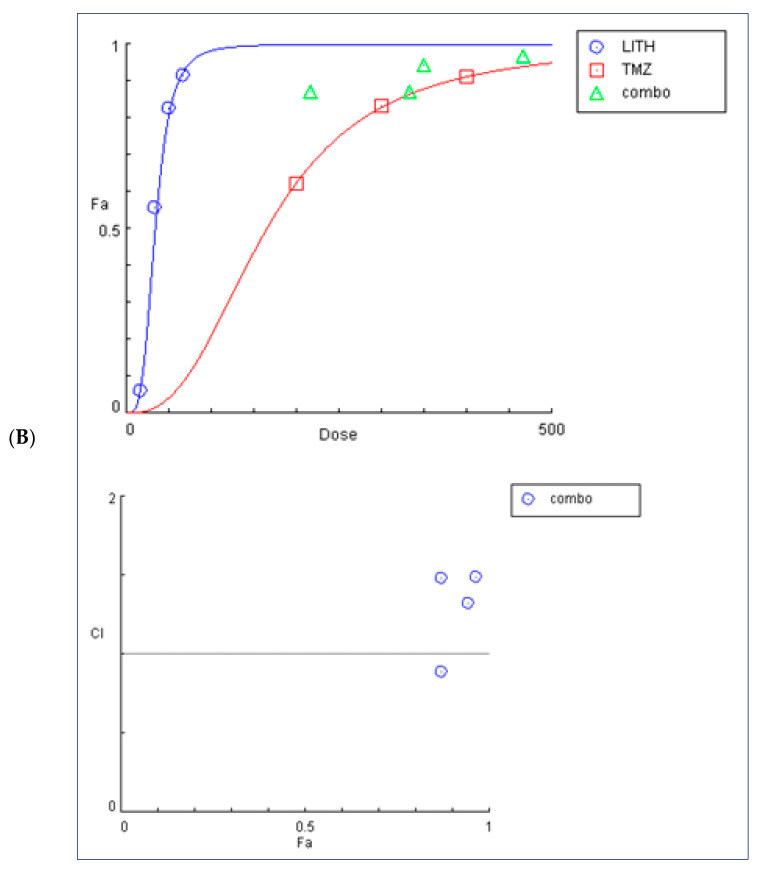
Graphs obtained from the CompuSyn report for the combination of 9″-lithospermic acid methyl ester and TMZ in (**A**) U87 and (**B**) T98 cells, depicting the dose–response curves and the combination–index graph.

**Figure 8:**
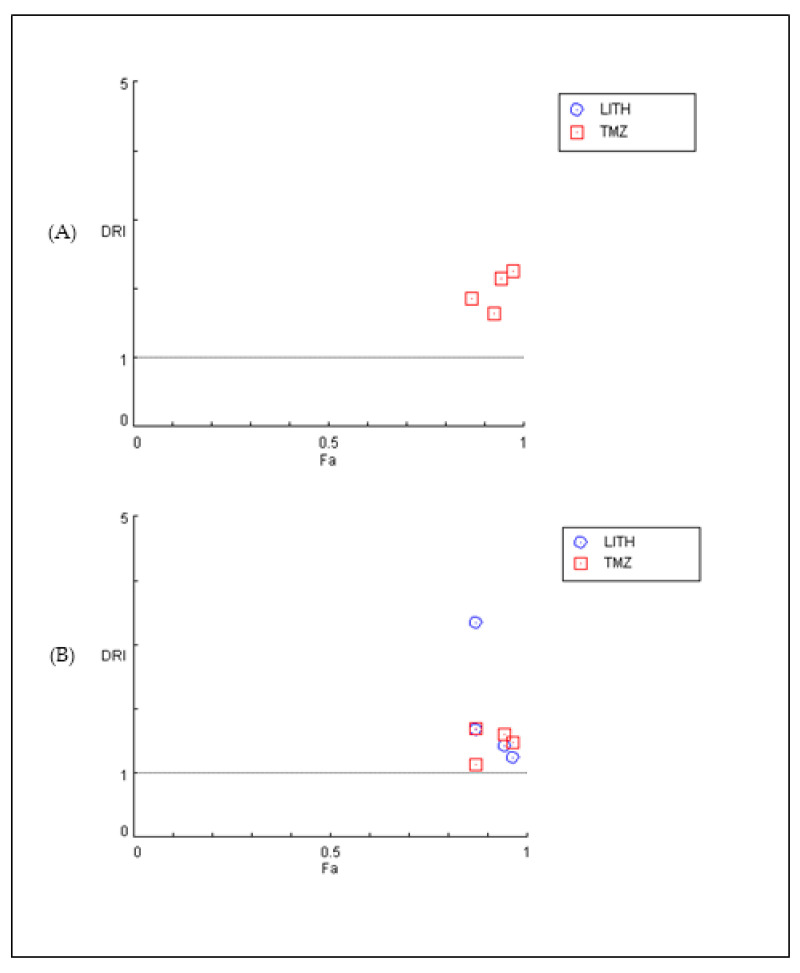
Dose reduction plots for the combination of 9″-lithospermic acid methyl ester and TMZ at different experimental sites in cell lines (**A**) U87 and (**B**) T98. DRI > 1 shows a favorable dose reduction of both agents. DRI > 5 values do not appear in the plot.

The graphical representation of the combined effect of 9″-lithospermic acid methyl ester and TMZ is also shown through dose–effect curves and combination–index plots generated using CompuSyn software for the U87 and T98 cell lines (Figure 7). For each combination of compounds, CompuSyn calculates the dose reduction index, where it can take values of DRI > 1 and <1, indicating favorable and unfavorable dose reduction, respectively, and DRI = 1, indicating no dose reduction. As shown in Figure 8 in both the U87 (A) and T98 (B) cell lines, combinations of the above substances show a favorable dose reduction (DRI > 1).

## 3. Discussion

Cancer is one of the leading causes of death worldwide. Extensive research and advances in technology have made a catalytic contribution to our knowledge of the biology and various mechanisms of cancer. The development of chemotherapeutic drugs, as well as the use of radiotherapy, have contributed significantly to increasing survival, but the adverse effects of these treatments and the recurrence of the disease limit their effectiveness. For these reasons, it is imperative that new, more effective drugs are discovered. So far, a plethora of active compounds from plants and herbs have been found with potential cytotoxic properties against various types of cancer [14,16,17,18,19]. For this reason, we undertook the study of a natural compound, 9″-lithospermic acid methyl ester, isolated from the methanolic extract of *Thymus thracicus* Velen or thyme. According to the literature, lithospermic acid possesses antioxidant and anti-inflammatory properties, the use of which has resulted in a preventive effect on the development of diabetic retinopathy, protection against oxidative damage in hepatocytes [20], and, finally, the amelioration of myocardial ischemia through a wide range of antioxidant mechanisms [21].

In the present study, we investigated the effect of 9″-lithospermic acid methyl ester on malignant cells and, more specifically, on the human glioblastoma cell lines U87 and T98. For this purpose, the agent diluted in DMSO was used and added at different concentrations, as well as for different time intervals, to determine the effect on cell viability and, in addition, to find the IC_50_ using Trypan Blue and Crystal Violet staining. The results showed a cytotoxic effect of the 9″-lithospermic acid methyl ester through a dose-dependent, but also time-dependent, mode. An attempt was then made to determine the effect on the cell cycle, where a cell arrest in the S phase and an accumulation of cells in the sub. G0/G1 phase was observed, indicating cell death. In wound healing simulation experiments, it was shown that the incubation of cells with 9″-lithospermic acid methyl ester reduces cell migration to satisfactory levels. Since the chemotherapeutic agent TMZ is the main treatment against glioblastomas, a combined incubation of cells with these two compounds was performed, and a synergy in cytotoxicity was observed.

In a previous study by Kohda et al., the methyl ester of lithospermic acid was found to inhibit adenylate cyclase, an enzyme responsible for the production of the second messenger cyclic AMP (cAMP), which plays a key role in cellular signaling [22]. cAMP-related signal transduction has been found to control the induction of apoptosis in different tumors, including GBM [23]. In particular, Friesen et al., showed that the downregulation of cAMP resulted in the radio- and chemo-sensitization of GBM cells to doxorubicin through the induction of apoptosis [24]. Based on these results, and the fact that 9″-lithospermic acid methyl ester increased cell death in the two GBM cell lines tested in our experiments, it is possible that its anti-glioma properties could partly be attributed to the induction of apoptosis.

These results show a remarkable activity of 9″-lithospermic acid methyl ester against glioblastoma cells. To the best of our knowledge, there are no other research papers examining the antineoplastic activity of this substance, except the study by F. Yang et al., 2022, which showed that the use of lithospermic acid methyl ester in a neuroblastoma cell line (SH-SY5Y) resulted in the inhibition of cell viability, with the IC_50_ being equal to 85.93 μΜ [15], while in our experiments, the IC_50_ was found to be equal to 30 μM and 34 μM in cell lines U87 and T98, respectively.

## 4. Materials and Methods

### 4.1. Isolation and Identification of 9″-Lithospermic Acid Methyl Ester

**Figure 9:**
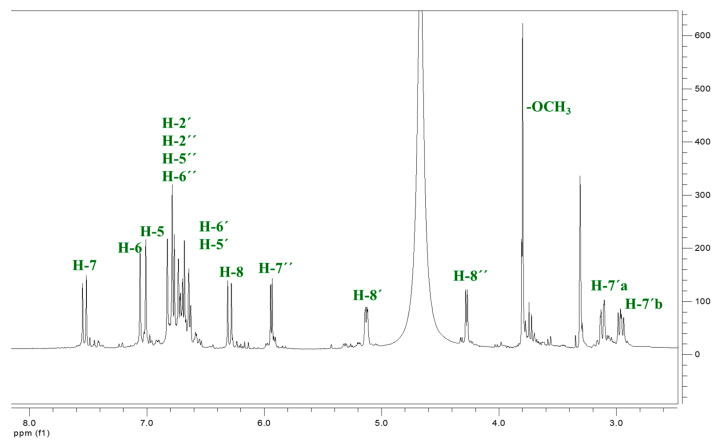
^1^H-NMR spectrum of 9″-methyl lithospermate (CD_3_OD, 500 MHz).

**Figure 10:**
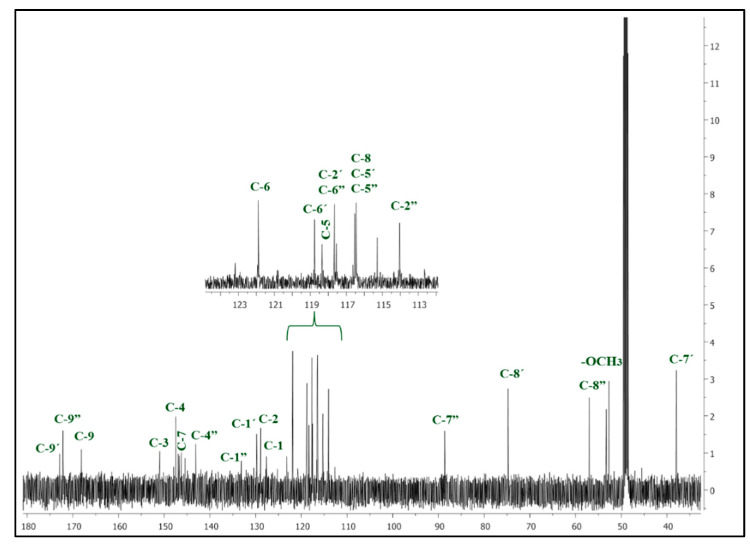
^13^C-NMR spectrum of 9″-methyl lithospermate (CD_3_OD, 125 MHz).

9″-Lithospermic acid methyl ester was derived from the methanol extract of *Thymus thracicus* Velen (Lamiaceae), collected from the wild from Mt. Paggaio in Northwest Greece. The plant was taxonomically identified by Dr. Nikos Krigas (Institute of Plant Breeding and Genetic Resources, Hellenic Agricultural Organization—Demeter, 57001 Thermi, Greece), and a voucher specimen has been deposited at the Herbarium of the School of Pharmacy (No Lazari D. 7348). Air-dried aerial parts of the plant (319.8 g) were exhaustively extracted with petroleum ether (P.E. 40–60°), dichloromethane (DM), and methanol (MeOH) at room temperature. Each of the individual extracts was subjected to evaporation process at a low temperature (40 °C) on a rotary distillation apparatus under vacuum (Rotavapor). The methanol soluble extract was submitted to the Charaux–Paris method [25] and then to consecutive extractions with ether, ethyl acetate, and n-butanol. The ethyl acetate fraction (4.19 g), which was chosen for further isolation procedures, was first submitted to column chromatography (CC) with methanol (100%) over Sephadex LH-20, yielding 13 fractions (A-M). Fraction E (225.2 mg) was subjected to silica column chromatography Silica 9385 (Merck Art. 9385, Darmstadt, Germany) and eluted with mixtures of DM/MeOH/H_2_O, yielding 14 fractions (EA-EN), from which the fraction EJ eluted with DM:MeOH:H_2_O 65:35:3.5 (55.0 mg) was identified as methyl lithospermate by means of NMR spectroscopy. Thin Liquid Chromatography (TLC) was used to control the quality of the fractions. For the TLC, a silica gel (Kieselgel F254, Merck, Art. 5554) stationary phase on aluminum foil (20 cm × 20 cm, 0.1 mm) with a fluorescence marker was used. The development of the TLC plates was carried out using mixtures of solvents appropriate for each group of fractions. Finally, the TLC plates were sprayed with vanillin-H_2_SO_4_ (1:1). The identification/verification of methyl lithospermate was performed via 1D and 2D NMR studies (^1^H, ^13^C, COSY, HSQC, and HMBC). The NMR spectra were recorded in CD_3_OD using an AGILENT DD2 500 (500.1 MHz for ^1^H-NMR and 125.5 MHz for ^13^C-NMR) spectrometer. Chemical shifts are reported in δ (ppm) values relative to TMS (3.31 ppm for ^1^H-NMR and 49.05 ppm for ^13^C-NMR for CD_3_OD). The data of isolated and identified methyl lithospermate (Table 3 and Figure 9 and Figure 10) were compared with those of samples from our collection and/or with reported data in the literature (Figures S1–S3) [22].

### 4.2. Cell Lines and Treatment Conditions

The human glioma U87 cell line was a gift from Dr W.K. Alfred Yung (Department of Neuro-Oncology, M.D. Anderson Cancer Center, Houston, TX, USA), and the human glioma cell line T98 was purchased from ATCC (Manassas, VA, USA). Both cell lines were cultured in Dulbecco’s Modified Eagle’s Medium (Gibco BRL, Life Technologies, Grand Island, NY, USA), supplemented with 10% fetal bovine serum (FBS) and 1% penicillin– streptomycin (100 µg/mL of streptomycin and 100 Units/mL of penicillin), Gibco BRL. The cell lines were incubated in a humidified atmosphere adjusted at 5% CO_2_ and 37 °C. 9″-lithospermic acid methyl ester isolated from the methanolic extract of *Thymus thracicus* Velen (thyme) was diluted in DMSO and stored at −80 °C. Temozolomide (TMZ) was obtained from Sigma Aldrich, diluted in DMSO, and stored at −20 °C. Cultures of T98 or U87 glioma cells were treated with 9″-lithospermic acid methyl ester alone or in combination with TMZ [7].

### 4.3. Trypan Blue Assay

The cell viability of 9″-lithospermic acid methyl ester was assessed using the Trypan Blue exclusion assay for both the U87 and T98 cell lines. Specifically, 10,000 cells were seeded in 24-well plates, and after 24 h of incubation, they were exposed to increasing concentrations of 9″-Lithospermic methyl ester (0–70 μM). The cells were incubated for an additional 72 h, and then Trypan Blue stain was added. The cell viability was determined at 72 h post-treatment utilizing phase contrast microscopy. The cytotoxicity assay was performed in triplicate, and the results represent the mean of the three assays. Additionally, the Trypan Blue exclusion assay was used to assess the viability of the cells following co-treatment with TMZ and 9″-lithospermic acid methyl ester [19].

### 4.4. Crystal Violet Assay

An additional test for cell viability, the Crystal Violet assay, was also utilized to calculate cell viability. Cells (10^5^) were seeded per well in six-well plates and, 24 h later, were treated with 9″-lithospermic acid methyl ester at concentrations of IC_50_ and doubled IC_50_, as previously calculated through the Trypan Blue Exclusion Assay (34 and 68 µM for the T98 cell line and 30 and 60 µM for the U87 cell line). The cells were incubated for 72 h, washed twice with phosphate-buffered saline (PBS), and then Crystal Violet solution 0.2% (0.2 g Crystal Violet Powder, MERCK in 80 mL ddH_2_O and 20 mL Methanol, MERCK GLOBAL, Athens, Greece) was added in each well and the plates were placed on a rocking shaker for 2–3 min. Subsequently, the plates were rinsed with running water twice and left to dry for 24 h, after which pictures of every well-plate were obtained by phase-contrast microscopy [19].

### 4.5. Flow Cytometric Analysis of DNA Cell Cycle

For flow cytometry, 10^4^ cells were treated with increased concentrations (34 and 68 µM for the T98 cell line and 30 and 60 µM for the U87 cell line) of 9″-lithospermic acid methyl ester. As a negative control, untreated cells containing less than 1% DMSO were employed. All samples were run in triplicate, and at least three separate experiments were conducted. Flow cytometric analysis was performed 72 h post-treatment with 9″-lithospermic acid methyl ester. For the DNA cell cycle, cells were treated with trypsin, centrifuged, washed with PBS twice, and then incubated with PI (Propidium Iodide) working solution (50 µg/mL PI, 20 mg/mL RNase A, and 0.1% Triton X-100, Sigma-Aldrich, St. Louis, MO, USA) for 15 min at 37 °C in the dark. With the use of a flow cytometer (Omnicyt Flow Cytometer, Cytognos, Athens, Greece), the PI fluorescence of 10^4^ individual nuclei was determined. Subsequently, the fractions of cells in G0/G1, S, G2/M, and sub-G0/G1 phases were analyzed [18].

### 4.6. Combination Treatment with 9″-Lithospermic Acid Methyl Ester and Temozolomide

Cells (10⁴) were seeded in 24-well plates and treated with increasing concentrations of either 9″-lithospermic acid methyl ester, temozolomide (TMZ), or a combination of both. Cell viability for every condition was evaluated using the Trypan Blue exclusion assay at 72 h post-treatment. T98 cells were treated with 9″-lithospermic acid methyl ester in concentrations of 17 μM, 34 μM, 51 μM, and 68 μM, while TMZ was added in concentrations 200 μM, 300 μM, and 400 μM. U87 cells were treated with 9″-lithospermic acid methyl ester in concentrations of 15 μM, 30 μM, 45 μM, and 60 μM and with TMZ in concentrations of 25 μM, 50 μM, and 100 μM. For both series, three replicates of each condition were analyzed. The combined effect of 9″-lithospermic acid methyl ester and TMZ was evaluated using the Chou–Talalay combination index method [26]. The Combination Index (CI) was determined using the CompuSyn software. The affected fraction (Fa) of cells after treatment with 9″-Lithospermic methyl ester alone, TMZ alone, or different combinations of those two was calculated, and the dose–effect curves were generated. The Combination Index (CI) was determined using CompuSyn software. The impact of the combination treatment is represented by the CI value. It has been determined that for CI < 1, the effect is synergistic, for CI = 1, it is additive, and for CI > 1, is antagonistic [27].

### 4.7. Scratch Wound Healing Assay

Cells (10⁵) were seeded in six-well plates and incubated in 10% FBS-containing medium for 24 h; then, the medium was replaced by another one containing 1% FBS (starving period) for an additional 24 h. Subsequently, the culture medium was changed again to a fresh medium containing 1% FBS, and a scratch wound was created by scraping each well from top to bottom, utilizing a 200 µL sterile pipette tip, followed by the addition of 9″-lithospermic acid methyl ester at concentrations of IC_50_ and 2IC_50_ (34 and 68 µM for the T98 cell line and 30 and 60 µM for the U87 cell line). Control cells followed the same procedures without adding the lithospermic acid methyl ester. Images of the cells that migrated to the cell-free scratch wound area were taken using phase contrast microscopy at the 0, 24, 48, and 72 h time marks post-treatment. The distance between the wound edges was measured using ImageJ software version 1.53t (National Institutes of Health, Bethesda, MD, USA) [18].

### 4.8. Statistical Analysis

All results are presented as the mean ± standard deviation. To determine the IC_50_ value of 9″-lithospermic acid methyl ester, the GraphPad Prism software (v. 8.0.0, San Diego, CA, USA, Trial Version) was used through non-linear regression analysis. A two-tailed Student’s *t*-test determined the results of the statistical analysis via Microsoft Excel (Microsoft 365). When *p* < 0.05, differences were deemed significant. The distance between the scratch edges was measured using ImageJ software.

## 5. Conclusions

In conclusion, 9″-lithospermic acid methyl ester may represent a potential candidate for the treatment of glioblastoma, since the use of a combination of laboratory techniques has shown that this agent can inhibit cell proliferation, lead to cell death, and inhibit cell motility, features that are common in these neoplasms. In addition, the combined use of the chemotherapeutic agent TMZ resulted in intensified cell death. Future goals include the broader investigation of this substance in more cell lines and confirmation of the above results with different techniques to assess if this compound represents a potential complementary anti-glioma therapy.

## Figures and Tables

**Table 1:** Distribution percentage of the different phases of the cell cycle after evaluation through flow cytometry. Cell populations at different cell cycle phases were quantified, and data were expressed as the mean ± SD from three different experiments.

	Agent Concentration	Sub. G0/G1	G0/G1	S	G2/M
U87	Control	0.7% ± 0.20%	75.1% ± 1.90%	18.1% ± 1.15%	3.4% ± 0.35%
	IC_50_	2.8% ± 0.25%	63.7% ± 1.10%	24.6% ± 3.53%	5.6% ± 1.22%
	2IC_50_	12.2% ± 1.00%	51.8% ± 1.59%	27.0% ± 2.52%	6.3% ± 0.61%
T98	Control	1.2% ± 0.26%	67.6% ± 1.34%	17.3% ± 1.01%	11.8% ± 0.06%
	IC_50_	14.1% ± 0.42%	44.2% ± 0.50%	23.6% ± 0.82%	13.2% ± 0.38%
	2IC_50_	29.3% ± 2.98%	30.5% ± 3.00%	23.8% ± 0.93%	11.1% ± 0.42%

**Table 2:** Evaluation of the combined effect of 9″-lithospermic acid methyl ester and the chemotherapeutical agent temozolomide in glioblastoma cell lines (Combination index plot (CI) = 1 defines an additive effect, CI < 1 defines synergism, and CI > 1 is antagonism).

**U87**				
**9″–LITH (μM)**	**TMZ (μM)**	**EFFECT**	**CI**	**RESULT**
30.0	50.0	0.9251	0.75558	SYNERGY
15.0	25.0	0.8682	0.64668	SYNERGY
45.0	50.0	0.9432	0.64868	SYNERGY
60.0	100.0	0.9742	0.58751	SYNERGY
**T98**				
**9″–LITH (μM)**	**TMZ (μM)**	**EFFECT**	**CI**	**RESULT**
34.0	300.0	0.8702	1.4817	ANTAGONISM
17.0	200.0	0.8702	0.8881	SYNERGY
51.0	300.0	0.9441	1.323	ANTAGONISM
68.0	400.0	0.9664	1.4882	ANTAGONISM

**Table 3:** ^1^H and ^13^C NMR of 9″-methyl lithospermate (CD_3_OD, 500 MHz).

No	δ_C_	Type C	δ_H_	H	J (Hz)
1	127.5	C	-	-	-
2	130.1	C	-	-	-
3	150.6	C	-	-	-
4	146.9	C	-	-	-
5	118.1	CH	7.01	1	*d* (J = 8.0)
6	121.9	CH	7.06	1	*d* (J = 8.0)
7	145.9	CH	7.54	1	d (J = 16.0)
8	116.3	CH	6.30	1	d (J = 16.0)
9	168.9	C=O	-	-	-
1′	131.2	C	-	-	-
2′	117.4	CH	6.78	1	br s
3′	146.6	C	-	-	-
4′	144.8	C	-	-	-
5′	116.5	CH	6.68	1	d (J = 8.5)
6′	118.8	CH	6.63	1	*br dd* (J = 8.5)
7′a	38.7	CH_2_	3.11	1	*br dd* (J = 13.7, 3.4)
7′b			2.96	1	*dd* (J = 13.7, 9.8)
8′	77.5	CH	5.13	1	br dd (J = 9.8, 3.4)
9′	177.4	C=O	-	-	-
1″	133.2	C	-	-	-
2″	114.1	CH	6.82	1	br s
3″	146.3	C	-	-	-
4″	143.0	C	-	-	-
5″	116.7	CH	6.77	1	d (J = 8.5)
6″	117.7	CH	6.72	1	dd (J = 8.5, 1.9)
7″	88.5	CH	5.94	1	d (J = 7.0)
8″	57.0	CH	4.28	1	d (J = 6.8)
9″	172.9	C=O	-	-	-
-OCH_3_	53.3	CH_3_	3.80	3	s

## Data Availability

The data underlying this article will be shared on reasonable request to the corresponding author.

## Supplementary Materials

The supporting information can be downloaded at: https://www.mdpi.com/article/10.3390/ijms25042094/s1.

## Abbreviations

CI	Combination Index
DMSO	Dimethyl Sulfoxide
FBS	Fetal Bovine Serum
GBM	Glioblastoma
HIV	Human Immunodeficiency Virus
PBS	Phosphate-Buffered Saline
PI	Propidium Iodide
PI3K	Phosphoinositide 3—kinase
TMZ	Temozolomide

## References

[1] Nabors L.B., Portnow J., Ahluwalia M., Baehring J., Brem H., Brem S., Butowski N., Campian J.L., Clark S.W., Fabiano A.J. (2020). Central Nervous System Cancers, Version 3.2020, NCCN Clinical Practice Guidelines in Oncology. J. Natl. Compr. Cancer Netw..

[2] Tamimi A.F., Juweid M. (2017). Epidemiology and Outcome of Glioblastoma. Glioblastoma [Internet].

[3] Ostrom Q.T., Gittleman H., Farah P., Ondracek A., Chen Y., Wolinsky Y., Stroup N.E., Kruchko C., Barnholtz-Sloan J.S. (2013). CBTRUS statistical report: Primary brain and central nervous system tumors diagnosed in the United States in 2006–2010. Neuro Oncol..

[4] Koshy M., Villano J.L., Dolecek T.A., Howard A., Mahmood U., Chmura S.J., Weichselbaum R.R., McCarthy B.J. (2012). Improved survival time trends for glioblastoma using the SEER 17 population-based registries. J. Neurooncol..

[5] Kyritsis A.P., Levin V.A. (2011). An algorithm for chemotherapy treatment of recurrent glioma patients after temozolomide failure in the general oncology setting. Cancer Chemother. Pharmacol..

[6] Stupp R., Mason W.P., van den Bent M.J., Weller M., Fisher B., Taphoorn M.J., Belanger K., Brandes A.A., Marosi C., Bogdahn U. (2005). Radiotherapy plus concomitant and adjuvant temozolomide for glioblastoma. N. Engl. J. Med..

[7] Lazari D., Alexiou G.A., Markopoulos G.S., Vartholomatos E., Hodaj E., Chousidis I., Leonardos I., Galani V., Kyritsis A.P. (2017). N-(p-coumaroyl) serotonin inhibits glioblastoma cells growth through triggering S-phase arrest and apoptosis. J. Neurooncol..

[8] Kelley C.J., Mahajan J.R., Brooks L.C., Neubert L.A., Breneman W.R., Carmack M. (1975). Polyphenolic Acids of *Lithospermicum ruderale* (Boraginaceae). I. Isolation and Structure Determination of Lithospermic Acid. J. Org. Chem..

[9] Ai C.B., Li L.N. (1988). Stereo structure of salvianolic acid B and isolation of salvianolic acid C from salvia miltiorrhiza. J. Nat. Prod..

[10] Murata T., Oyama K., Fujiyama M., Oobayashi B., Umehara K., Miyase T., Yoshizaki F. (2013). Diastereomers of Lithospermic acid and Lithospermic acid B from *Monarda fistulosa* and *Lithospermicum erythrorhizon*. Fitoterapia.

[11] Abd-Elazem I.S., Chen H.S., Bates R.B., Huang R.C.C. (2002). Isolation of two highly potent and non-toxic inhibitors of human immunodeficiency virus type 1 (HIV-1) integrase from *Salvia miltiorrhiza*. Antivir. Res..

[12] Murata T., Miyase T., Yoshizaki F. (2010). Cyclic Spermidine Alkaloids and Flavone Glycosides from *Meehania fargesii*. Chem. Pharm. Bull..

[13] Tanaka T., Moromoto S., Nonaka G., Nishioka I., Yokazawa T., Chung H.Y., Oura H. (1989). Magnesium and Ammonium-Potassium Lithospermicates B, the Active Principles Having a Uremia-Preventive Effect from *Salvia miltiorrhiza*. Chem. Pharm. Bull..

[14] Jin C.J., Yu S.H., Wang X.M., Woo S.J., Park H.J., Lee H.C., Choi S.H., Kim K.M., Kim J.H., Park K.S. (2014). The Effect of Lithospermic Acid, an Antioxidant, on Development of Diabetic Retinopathy in Spontaneously Obese Diabetic Rats. PLoS ONE.

[15] Yang F., Chen Z.R., Yang X.H., Xu Y., Ran N.J., Liu M.J., Jin S.G., Jia H.N., Zhang Y. (2022). Monomethyl Lithospermicate alleviates ischemic stroke injury in middle cerebral artery occlusion mice in vivo and protects oxygen glucose deprivation/reoxygenation induced SHSY-5Y cells in vitro via activation of PI3K/Akt signaling. Front. Pharmacol..

[16] Greenwell M., Rahman P.K.S.M. (2015). Medicinal Plants: Their Use in Anticancer Treatment. Int. J. Pharm. Sci. Res..

[17] Khan T., Ali M., Khan A., Nisar P., Jan S.A., Afridi S., Shinwari Z.K. (2019). Anticancer Plants: A Review of the Active Phytochemicals, Applications in Animal Models, and Regulatory Aspects. Biomolecules.

[18] Zoi V., Papagrigoriou T., Tsiftsoglou O.S., Alexiou G.A., Giannakopoulou M., Tzima E., Tsekeris P., Zikou A., Kyritsis A.P., Lazari D. (2023). Therapeutic Potential of Linearol in Combination with Radiotherapy for the Treatment of Glioblastoma In Vitro. Int. J. Mol. Sci..

[19] Giannakopoulou M., Dimitriadis K., Koromili M., Zoi V., Vartholomatos E., Galani V., Kyritsis A.P., Alexiou G.A., Lazari D. (2022). Siderol Inhibits Proliferation of Glioblastoma Cells and Acts Synergistically with Temozolomide. Biomedicines.

[20] Chan K.W.K., Ho W.S. (2015). Anti-oxidative and hepatoprotective effects of lithospermic acid against carbon tetrachloride-induced liver oxidative damage in vitro and in vivo. Oncol. Rep..

[21] Zhang M., Wei L., Xie S., Xing Y., Shi W., Zeng X., Chen S., Wang S., Deng W., Tang Q. (2021). Activation of Nrf2 by Lithospermic Acid Ameliorates Myocardial Ischemia and Reperfusion Injury by Promoting Phosphorylation of AMP-Activated Protein Kinase α (AMPKα). Front. Pharmacol..

[22] Kohda H., Takeda O., Tanaka S., Yamasaki K., Yamashita A., Kurokawa T., Ishibashi S. (1989). Isolation of inhibitors of adenylate cyclase from dan-shen, the root of *Salvia miltiorrhiza*. Chem. Pharm. Bull..

[23] Insel P.A., Zhang L., Murray F., Yokouchi H., Zambon A.C. (2012). Cyclic AMP is both a pro-apoptotic and anti-apoptotic second messenger. Acta Physiol..

[24] Friesen C., Hormann I., Roscher M., Fichtner I., Alt A., Hilger R., Debatin K.M., Miltner E. (2014). Opioid receptor activation triggering downregulation of cAMP improves effectiveness of anti-cancer drugs in treatment of glioblastoma. Cell Cycle.

[25] Paris R., Nothis A. (1970). Plantes médicinales et Phytothérapie. Tome IV.

[26] Chou T.C. (2006). Theoretical Basis, Experimental Design, and Computerized Simulation of Synergism and Antagonism in Drug Combination Studies. Pharmacol. Rev..

[27] Chou T.C. (2010). Drug Combination Studies and Their Synergy Quantification Using the Chou-Talalay Method. Cancer Res..

