# Supplementary material for: Antineoplastic Activity of 9″-Lithospermic Acid Methyl Ester in Glioblastoma Cells
Source: Int J Mol Sci. 2024 Feb 8;25(4):2094. doi: 10.3390/ijms25042094 (PMC10889145; doi:10.3390/ijms25042094)
Supplement: Supplementary file 1 [file ijms-25-02094-s001.zip › ijms-2808323-supplementary.pdf]

SUPPLEMENTARY MATERIAL

Antineoplastic Activity of 9′′–Lithospermic Acid Methyl Ester in Glioblastoma Cells

Panagiota Tzitiridou[1], Vasiliki Zoi[1], Theodora Papagrigoriou[2], Diamanto Lazari[2], Chrissa Sioka[1], Georgios A. Alexiou[1,3], Athanassios P. Kyritsis[1]

[1]Neurosurgical Institute, University of Ioannina, 45500 Ioannina, Greece

[2]Laboratory of Pharmacognosy, Division of Pharmacognosy-Pharmacology, School of Pharmacy, Faculty of Health Sciences, Aristotle University of Thessaloniki, 54124 Thessaloniki, Greece

[3]Department of Neurosurgery, University of Ioannina, 45500 Ioannina, Greece

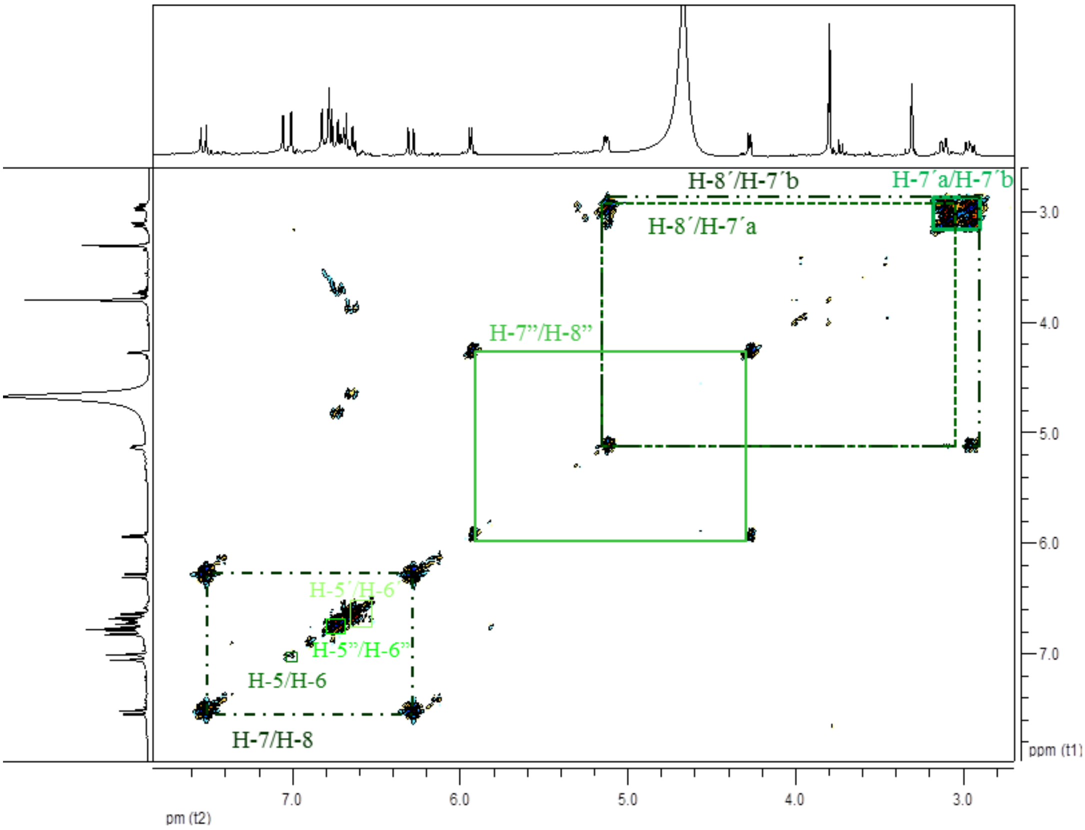

Figure S1. COSY Spectrum of 9′′-methyl lithospermate (CD_3OD, 500MHz).

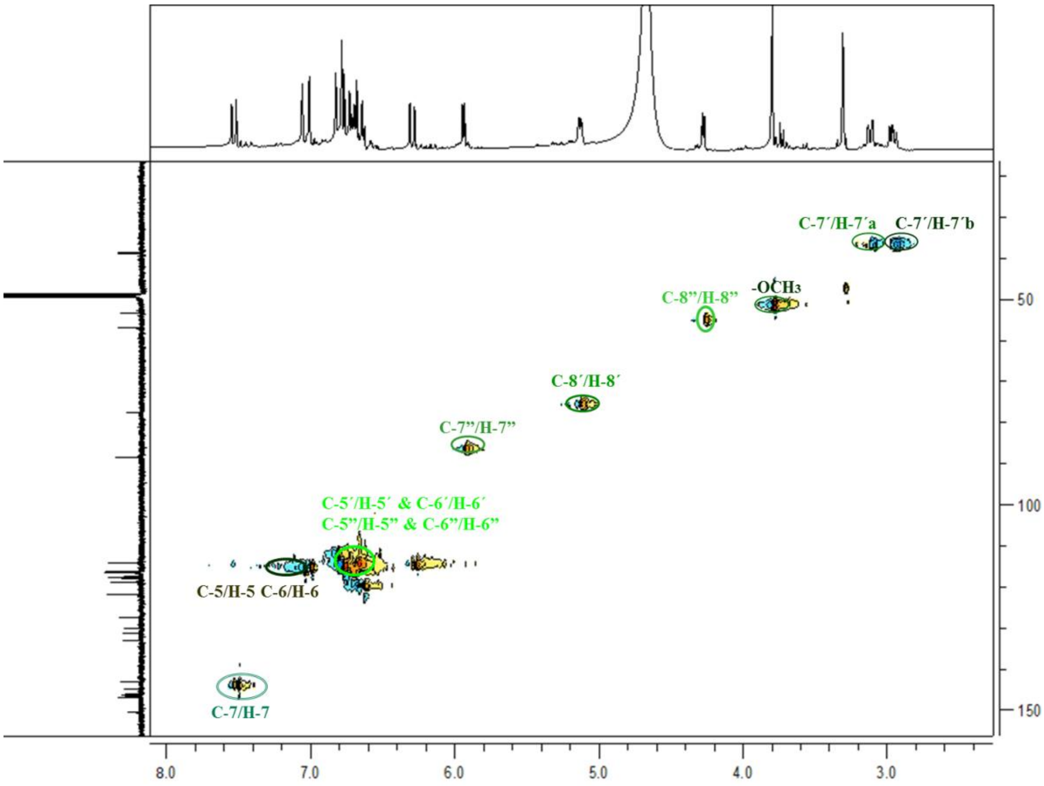

Figure S2. HSQC spectrum of 9″-methyl lithospermate (CD_3OD, 500MHz).

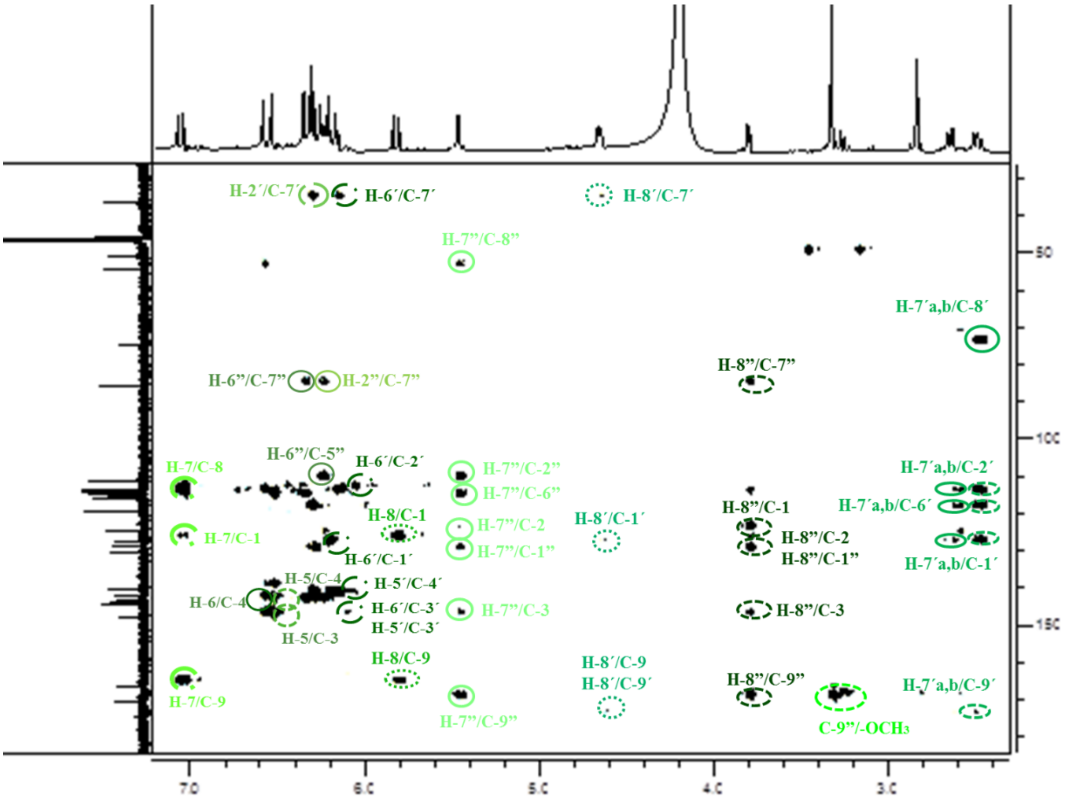

Figure S3. HMBC spectrum of 9″-methyl lithospermate (CD_3OD, 500MHz).
